# Preparation and Performance Research of Pb(II)-Imprinted Acrylonitrile-Co-Acrylic Acid Composite Material with Modified Sand Particles as Carrier

**DOI:** 10.3390/polym17020229

**Published:** 2025-01-17

**Authors:** Yixin Sui, Shuaibing Gao, Jiaxiang Qi, Shawket Abliz, Linlin Chai

**Affiliations:** Key Laboratory of Oil and Gas Fine Chemicals of Ministry of Education, School of Chemical Engineering, Xinjiang University, Urumqi 830017, China; suiyixin799@163.com (Y.S.); gsb1215@163.com (S.G.); qjx17613720518@163.com (J.Q.)

**Keywords:** ion-imprinted composites, sand particles, lead, adsorption

## Abstract

Lead (Pb) is classified as a prevalent metallic pollutant, significantly impacting the ecological environment, especially human health. Consequently, it is crucial to develop adsorbent materials that are environmentally friendly, cost-effective, and which possess high selectivity. This study aims to fabricate a Pb(II)-imprinted acrylonitrile-co-acrylic acid composite material by using modified sand particles as the carrier, and then to investigate its properties. Through pretreatment of sand particles, acrylonitrile and acrylic acid were polymerized on the surface of modified sand particles, and Pb(II) served as a template ion for imprinting. A variety of characterization methods were used to verify the composite material and conduct an analysis of its morphology, chemical composition, and pore characteristics. The adsorption efficiency of this composite material for Pb(II) is comprehensively explored, with the process involving adsorption kinetics, adsorption isotherms, selective adsorption, and reuse experiments. Through static adsorption experiments, multiple elements influencing the adsorption ability of the composite material towards Pb(II) are investigated. It was demonstrated by the results that the composite material prepared possesses a rich pore structure and excellent Pb(II) recognition ability. The investigation on adsorption kinetics is in line with the quasi-first-order and quasi-second-order kinetic models, while the adsorption isotherm, obeys the Langmuir model. The ideal adsorption conditions were pH = 7, with the adsorption reaching equilibrium within 105 min. Even when multiple interfering ions were present, it still had high selectivity for Pb(II). The composite material showed an adsorption saturation capability reaching 41.83 mg·g^−1^, considerably surpassing the non-imprinted counterpart. After being reused eight times, the composite material can still maintain an adsorption efficiency for Pb(II) that is above 79% and demonstrates high potential in the practical application environment.

## 1. Introduction

In the context of the contemporary environmental crisis, the contamination of the planet’s ecosystems by heavy metals has emerged as a significant global challenge [1]. Lead (Pb(II)) is of particular concern as it is widely distributed in industrial wastewater, soil, and groundwater and is regarded as among the most prominent poisonous and carcinogenic heavy metal contaminants [2,3]. Contamination of the environment by Pb(II) is a pervasive phenomenon that can exert significant detrimental effects on the structure and functionality of ecosystems. These effects may manifest as the destruction of soil fertility and disruption of the survival and reproductive processes of aquatic organisms. Additionally, the contamination of food sources through the food chain has the potential to introduce Pb(II) into the human body [4], resulting in considerable harm to human health. In extreme instances, the condition may prove to be life-threatening [5]. This can manifest as damage to the nervous system, hematopoietic system, immune system, and kidneys, among others [6,7]. Furthermore, children’s intellectual capacity may be particularly vulnerable to the adverse effects of Pb(II) pollution [8,9,10]. Consequently, it is crucial to fabricate adsorbent materials that are highly efficient, economically viable, and which possess high specificity for eliminating Pb(II) from the environment [11].

At present, the principal methods for the elimination of Pb(II) are adsorption [12,13], chemical precipitation [14], ion exchange [15], biological method [16,17], and membrane separation [18]. The adsorption method holds substantial potential for application in the area of pollution control on account of multiple advantageous features, such as the simplicity of its operation, its cost-effectiveness, its high efficiency, and its ability to achieve selective adsorption [19,20]. Adsorbent materials represent a crucial means of efficient Pb(II) removal and exhibit a vast array of forms and compositions. These include natural materials [21], modified materials [22], metal–organic skeleton materials [23], nanomaterials [24], and biosorbent materials [25]. Adsorbent materials with diverse properties present a variety of approaches for effectively eliminating Pb(II) in the environment and act as an indispensable means for safeguarding the environment and human health. Ion-imprinted polymers(IIPs) [26] technology is emerging as a prominent solution in the ongoing efforts to address the escalating issue of heavy metal contamination.

**Figure 1:**
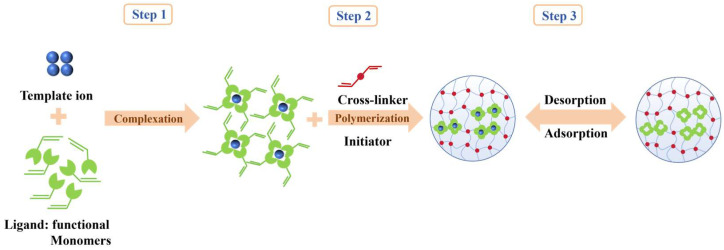
The preparation of IIPs.

Ion-imprinted polymers, which represent an extension of molecular-imprinted (MIPs), constitute an advanced technology that specializes in the recognition and adsorption of specific ions [27]. The target ion serves as a template for the formation of polymers with specific recognition sites in the presence of crosslinking agents [28]. This process occurs through specific interactions with functional monomers, including ligand interactions and electrostatic attractions. As illustrated in Figure 1, upon elution of the template ions, imprinted cavities are left in the polymer that exhibit a high degree of spatial structural and similar binding properties for the target ions [29]. These cavities serve as highly selective and efficient traps for the target ions, enabling their recognition and adsorption in complex environments. Their capacity to accurately capture target ions, despite the existence of various interfering ions, significantly enhances the removal efficiency and selectivity of metal ions. Xu et al. [28] selected N-Methylol acrylamide and AA as the functional monomers, utilized homemade chitin particles as the solid particles, and took EGDMA as the crosslinking agent to synthesize ruthenium temperature-sensitive ion-imprinted polymer microspheres. Shamsabadi, E. et al. [30] adopted a bulk polymerization approach for preparation. In this process, MAA served as the functional monomer, EGDMA was used as the crosslinking, phenyl morpholine-4-carbodithioic acid phenyl ester played the role of the chelating agent, and ammonium persulfate acted as the initiator. René et al. [31] used a fluorescent probe for Pb(II) grounded on coupled anthracene with 5-amino-8-hydroxyquinoline and styrene fraction as functional monomers. IIP and NIP microbeads were synthesized via precipitation polymerization in a DMSO-2-methoxy ethanol (1:1, *v*/*v*) mixture with EGDMA as crosslinking.

Ion-imprinted adsorbent materials represent a novel class of materials with distinct selective adsorption capabilities [32]. The selection of an appropriate carrier preparation can significantly influence the performance and practical utility of the resulting material. Over the past few years, substantial focus has been placed on the preparation of ion-imprinted polymers on the surface of carriers by means of surface imprinting techniques. Employing natural materials as carriers has been investigated as a way to augment the quantity of effective adsorption sites, thus increasing the specific surface area and enabling quicker recognition of target ions. This has the potential to improve the adsorption efficiency. In the past decade, silica-based materials have undergone significant modifications and alterations to enhance their adsorption capacity for heavy metal ions [33].

The sand particles are primarily composed of silica. Among the various silicon-based porous materials, mesoporous silica is the most prevalent [34]. Silicon dioxide is characterized by a highly developed pore structure that facilitates increased contact between the adsorbent and other adsorbents. Additionally, the sand particle surface contains a substantial number of Si-OH groups, which can undergo a range of chemical modifications. These modified silica surfaces can selectively adsorb specific substances. When sand particles serve as carriers of ion-imprinted adsorbent materials, the chemical stability of silica ensures that, even when confronted with a multitude of complex adsorbate solution systems, the carrier itself remains unaltered by chemical reactions, thereby preserving the effectiveness of the ion-imprinted sites and guaranteeing the structural soundness throughout the entire adsorbent material, which is crucial for its proper functioning.

In this paper, we present a novel method for the preparation of Pb(II)-imprinted polymers with enhanced chemical stability. The unique feature of our approach is the use of sand modified with γ-aminopropyltriethoxysilane (KH550) as a carrier. This choice not only provides a unique structural support but also contributes to the overall stability of the final product. The selection of acrylic acid (AA) and acrylonitrile (AN) as the functional monomers and Pb(II) as the template ion was carefully designed to optimize the blotting effect. First, Pb(II) was made to react with acrylonitrile and acrylic acid, and then crosslinked with ethylene glycol dimethacrylate (EGDMA) using potassium persulfate (KPS) as an initiator. Finally, the template Pb(II) was removed with hydrochloric acid to obtain an imprinted porous surface. The significance of this work lies in the potential applications of these Pb(II)-imprinted polymers. They can be effectively used in environmental monitoring for the selective extraction and detection of trace Pb(II) ions in complex matrices, which is essential for the protection of the environment and human health.

## 2. Experimental Materials and Procedures

### 2.1. Experimental Chemicals

The sand particles were collected from the Taklimakan Desert in Xinjiang, China. Shanghai Macklin Biochemical Technology Co. (Shanghai, China) was the source from which lead nitrate (Pb(NO_3_)_2_) and ethylene glycol dimethacrylate (EGDMA) were acquired. Tianjin Zhiyuan Chemical Reagent Co. (Tianjin, China) supplied potassium persulfate (K_2_S_2_O_8_), nickel chloride (NiCl_2_), zinc chloride (ZnCl_2_), and cupric sulfate (CuSO_4_·5H_2_O). Sodium hydroxide (NaOH) came from Tianjin Xinbote Chemical Co. (Tianjin, China) as well. Tianjin Guangfu Fine Chemical Research Institute Co. (Tianjin, China) was the provider for acrylonitrile (AN), and acrylic acid (AA) was procured from Tianjin Fuyu Fine Chemical Co. (Tianjin, China). Nanjing Chuangshi Chemical Auxiliary Co., Ltd. (Nanjing, China) was the source from which γ-aminopropyltriethoxysilane (KH550) was acquired. Urumqi Dicheng Chemical Co. (Urumqi, China) was where hydrochloric acid (HCl) was purchased. Deionized water was used to prepare all the solutions.

### 2.2. Experimental Apparatus

To observe the surface morphology, scanning electron microscopy (SEM) (ZEISS Sigma 300, Oberkochen, Germany) was employed. For the measurement of the X-ray photoelectron spectra (XPS), an X-ray photoelectron spectrometer (ESCALAB 250 Xi, Thermo Fisher Scientific, Waltham, MA, USA) was utilized. The BET (Brunauer–Emmett–Teller) analysis was carried out by means of a surface area and porosity analyzer (ASAP2460, Micromeritics, Norcross, GA, USA). Meanwhile, with the help of a Fourier transform infrared spectrometer (FTIR) (BRUKER VERTEX 70, Bruker Optics, Ettlingen, Germany), the infrared spectra of Pb(II)-AA-AN and Pb(II)-AA-AN were acquired. The UV–vis spectra of Pb(II), AA, AN, and Pb(II)-AA-AN were tested using an ultraviolet absorption spectrometer (Puxi T6, New Century, Dalian, China).

### 2.3. Synthesis of Sand Particles/Pb(II)-Imprinted Composite Material

#### 2.3.1. Sand Particles’ Pretreatment

In this study, sand particles from disparate locations within the Taklamakan Desert (Xinjiang, China) were selected as experimental materials. Firstly, to ensure the particle size of the sand particles, a 200-mesh sieve was used to screen them. Following screening, the sand particles were washed with deionized water to remove any water-soluble impurities that may have accumulated on the surface. This cleaning process was repeated until the deionized water and sand particles reached a nearly transparent state, thereby ensuring that the sand particles’ surface impurities were effectively removed. Subsequently, the cleaned sand particles were placed on a magnetic stirrer for 2 h, during which time the magnetic substances contained in the sand particles were removed. Subsequently, the sand particles grains were subjected to repeated cleansing until the cleaning solution became transparent. They were then transferred to an oven maintained at a temperature of 80 °C for expeditious drying. Subsequently, the sand particles were placed in a well-ventilated location and allowed to dry naturally until they reached room temperature, at which point the next operation was initiated. The cleaned sand particles were then immersed in a 1:9 mixture of KH550 and deionized water at room temperature. Given that the mixture was stratified and KH550 was not readily soluble, it was essential to place the mixture on a magnetic stirrer for agitation and allow the sand particles to soak in the mixture for 15 h. This facilitates the modification of sand particles by KH550, resulting in the formation of modified sand particles (MS). At the end of the immersion, the MS were dried in an oven at 60 °C for 6 h.

#### 2.3.2. Preparation of Pb(II)-AA-AN Complex

We weighed out 0.5 g of Pb(NO_3_)_2_ and dissolved it in 50 mL of deionized water. Then, 0.435 mL of AA and 0.32 mL of AN were added sequentially. The solution was constantly agitated at 30 °C for 2 h to promote the cooperation of Pb(II) with AA and AN, thereby forming the Pb(II)-AA-AN complex. Finally, the complex solution was examined using UV–vis to determine its absorbance.

#### 2.3.3. Preparation of MS/Pb(II)-IIP and MS/Pb(II)-NIP

**Figure 2:**
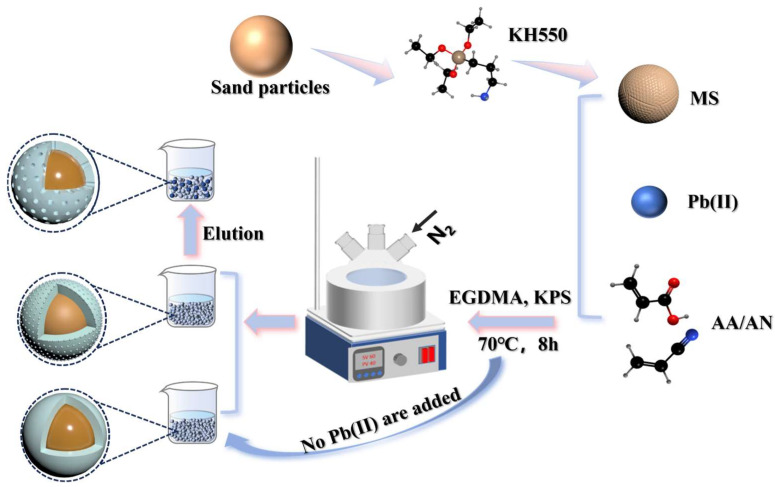
The process for preparing the polymer.

Precipitation polymerization was employed to synthesize Pb(II) ion-imprinted polymers (MS/Pb(II)-IIP). The previously synthesized Pb(II)-AA-AN complex was transferred to a three-necked round-bottomed flask, to which 5 g of silane-alkylated sand particles were added. The flask was then subjected to ultrasonication for 10 min in an ultrasonic device to ensure homogeneous mixing of the substances. Subsequently, 5.98 mL of the crosslinking agent EGDMA was introduced into the flask, and nitrogen was introduced into the reaction chamber for deoxygenation. In ion-imprinted polymerization, oxygen functions as a polymerization blocker, reacting with free radicals in the reaction system and impeding polymerization. Nitrogen gas can effectively remove oxygen from the reaction system, prevent interference, and ensure smooth polymerization [35]. The magnets were introduced into the flask, which was sealed, evacuated, and replaced with nitrogen. When the temperature climbed to 70 °C, we added 0.098 g of the initiator (KPS). Subsequently, the reaction was allowed to proceed within a nitrogen environment for 8 h until the polymerization was completed. The reaction-completed polymer was then removed and washed with deionized water to remove any unreacted monomer, crosslinker, or other components [36]. To eliminate the template ions, the composite material underwent repeated washings using 1 mol·L^−1^ HCl. The washings were carried out until the flame atomic absorption spectroscopy (FAAS) failed to detect any Pb(II) signal [37]. Then it was rinsed with distilled water several times until the pH became neutral. Subsequently, the wet polymer powder was placed within a drying oven at the temperature of 80 °C. Under the condition of not adding Pb(II), the non-imprinted polymer composite material (MS/Pb(II)-NIP) was prepared in the same way. The process for preparing the polymer is shown in Figure 2. After removing template ions, the solution containing Pb(II) was professionally treated by chemical precipitation method. Sodium sulfide was chosen as the precipitant to be added slowly and dropwise to the solution containing Pb(II), so that the Pb(II) in the solution existed in the form of black lead sulfide precipitates, which were separated by filtration. The lead sulfide precipitate is classified as hazardous waste, and we dispose of it appropriately, according to strict laboratory waste disposal specifications.

### 2.4. Adsorption Investigations

#### 2.4.1. Static Adsorption Investigations

To evaluate the adsorption behavior of Pb(II) in MS/Pb(II)-IIP materials, the adsorption properties were investigated through static adsorption experiments, and a series of Pb(II) solutions with diverse pHs were prepared and tested for the adsorption of Pb(II) at different temperatures and different Pb(II) aqueous concentrations. Adsorption times of 15, 30, 45, 60, 75, 90, 105, and 120 min were applied to test the adsorption of MS/Pb(II)-IIP. An amount of 0.1 mol·L^−1^ HCl or 0.1 mol·L^−1^ NaOH aqueous solution was used to adjust the pH, and MS/Pb(II)-IIP was added to the Pb(II) solution for adsorption. We centrifuged the samples and analyzed them at a specific time. The adsorption capacity *Q_t_* (mg·g^−1^), adsorption rate *E_t_*, and desorption rate *B_t_* of MS/Pb(II)-IIP at a specific time were calculated using the following equations [38]:

(1) $$Q_{t}=\frac{\left(C_{0}-C_{t}\right)V}{w}$$

(2) $$E_{t}=\frac{C_{0}-C_{t}}{C_{0}}\times 100\%$$

(3) $$B_{t}=\frac{C_{d}V_{d}}{\left(C_{0}-C_{t}\right)V}\times 100\%$$

where *C*_0_ (mg·L^−1^) is the initial concentration of Pb(II), *C_t_* (mg·L^−1^) is the Pb(II) concentration after adsorption for a particular time, *C_d_* (mg·L^−1^) is the desorption concentration of the Pb(II), *V* (L) is the volume of the Pb(II), *V_d_* (L) is the volume of the desorption solution, and w (g) is the weight of the dry adsorbate.

#### 2.4.2. Selectivity Adsorption

To examine the selectivity of MS/Pb(II)-IIP for Pb(II), a multi-ion static adsorption experiment was employed. We selected competing ions, like Zn(II), Cu(II), and Ni(II), that had the same charge and a radius of similar magnitude to Pb(II) [39]. The calculation formula for the parameters of the selectivity experiment is as follows, Equations (4)–(6) [40]:

(4) $$D=\frac{\left(C_{0}-C_{e}\right)V}{C_{0}m}$$

(5) $$\alpha =\frac{D_{Pb\left(II\right)}}{D_{M\left(II\right)}}$$

(6) $$k=\frac{\alpha_{MS/Pb(II)-IIP}}{\alpha_{MS/Pb(II)-NIP}}$$

where *D* is the distribution ratio, *V* is the volume of the solution (mL), m is the mass of the polymer (g), *C*_0_ and *C_e_* are the original and terminal concentrations of ions (mg·L^−1^), M(II) is the competing ion, α is selection coefficient, and *k* is coefficient of relative selection, respectively.

#### 2.4.3. Reuse Experiment

For adsorbents in industrialized practical applications, their stability and reusability serve as crucial criteria for evaluating their efficacy. The reuse of MS/Pb(II)-IIP can lead to a reduction in treatment costs and minimization of waste generation. When it comes to wastewater treatment, the reuse of an adsorbent material eliminates the need for frequent replacement, thereby conserving resources. To this end, a reproducible adsorption–desorption experiment was conducted using saturated MS/Pb(II)-IIP under optimized experimental conditions. This was carried out to measure the amount of Pb(II) present in the solution prior to and following adsorption, as well as to assess the reusability performance of MS/Pb(II)-IIP.

## 3. Results and Discussion

### 3.1. Characterization Investigations

#### 3.1.1. Contact Angle Measurement

**Figure 3:**
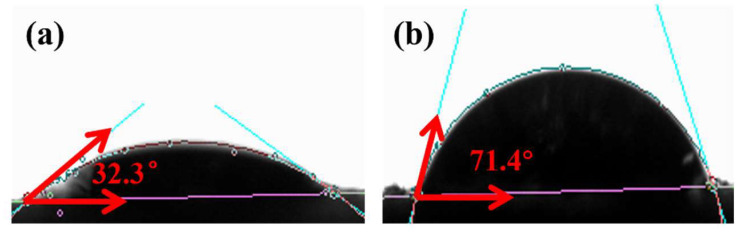
Contact angle measurement of sand particles (**a**) and modify sand particles (**b**).

The surface of sand particles typically contains some Si-OH. The chemical composition and surface properties of these particles determine their relatively specific surface energy and wettability. KH550 is a common silane coupling agent whose molecular structure contains both hydrolyzable alkoxyl (e.g., methoxyl, ethoxyl, etc.) and other organic functional groups (e.g., amino). These groups undergo hydrolysis and condensation when interacting with natural sand particles. Hydrolysis of the alkoxy group of KH550 forms a Si-OH, which can condense with the existing silicone hydroxyl group on the surface of sand particles. This results in the KH550 molecule being grafted onto the surface of sand particles through chemical bonding. The organic groups, such as alkyl chains, at the other end of the KH550 molecule will extend outward on the surface of sand particles, thereby modifying the surface of the sand particles. This process transforms the natural sand into alkylated sand particles. The modification results in a notable alteration, as evidenced by the contact angle test results illustrated in Figure 3. From Figure 3a, the contact angle θ = 32.3° for the natural sand particles. Given that alkyl chains are hydrophobic groups, the solid surface transforms its intrinsic nature, and this is accompanied by a reduction in surface energy following the attachment of a considerable number of alkyl chains to the surface of the sand particles. This results in an increase in the contact angle (θ), as evidenced by the modified sand particles in Figure 3b, which exhibit a contact angle of θ = 71.4°. The surface of the sand particles underwent a transition from a relatively hydrophilic to a relatively non-hydrophilic state, which is indicative of the impact of alkylation modification on the wettability of the sand particles’ surface. The alteration in the contact angle provides a clear representation of the change in the properties of the natural sand particles following KH550 modification.

#### 3.1.2. UV–Vis Adsorption Spectra

**Figure 4:**
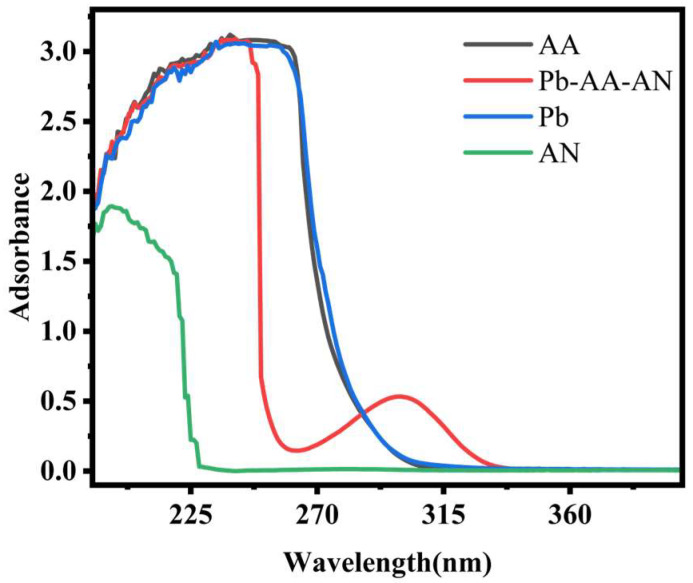
UV–vis spectra of Pb(II), AN solution, AA solution, and Pb(II)-AA-AN.

To investigate the interaction of Pb(II) with AA and AN, a UV–visible spectrophotometer was used. The UV–vis absorption spectra of Pb(II), AA, AN, and Pb(II)-AA-AN complexes are displayed in Figure 4. If Pb(II) binds to AA and AN without any coordination effect, the UV absorption spectrum should just show Pb(II) with AA and AN, with no new absorption peaks. This is because of the superposition principle of absorption spectra. But when Pb(II) is mixed with AA and AN, new absorption peaks show up in the UV spectrum. This shows that Pb(II) is coordinating with AA and AN. The reason for this phenomenon may be that Pb(II) has vacant electron orbitals, while the carboxy oxygen atom in AA and the nitrogen atom in AN are electronegative and present lone pairs of electrons with high electron cloud density. Coordination bonds can interconnect these electrons. As a result, the electron cloud density of the double bond in AA and the triple bond in AN is reduced and, under UV irradiation, the electron leaps can produce absorption spectra with lower energies, thus showing the redshift of the complexes in the long wavelength region. There is a significant broadening of the absorption peaks of Pb(II)-AA-AN near 270 nm, which may be due to the coordination interactions between Pb(II), AA, and AN. The coordination process broadens and widens the absorption peaks of the complexes. The coordination process alters the relatively independent and regular electronic structures of the components and is accompanied by redistribution and hybridization of the electron cloud. Specifically, the coordination of Pb(II) with AA and AN introduces more different electronic energy states, which allows the electron leaps to occur in a wider energy range, leading to the broadening of the absorption peaks. The interaction of the empty orbitals of Pb(II) with the lone electron pairs of AA and AN forms coordination bonds, which provide more leaps with the electrons in more pathways and energy states, thus increasing the probability of the electrons leaping between the different orbitals.

#### 3.1.3. FTIR Analysis

**Figure 5:**
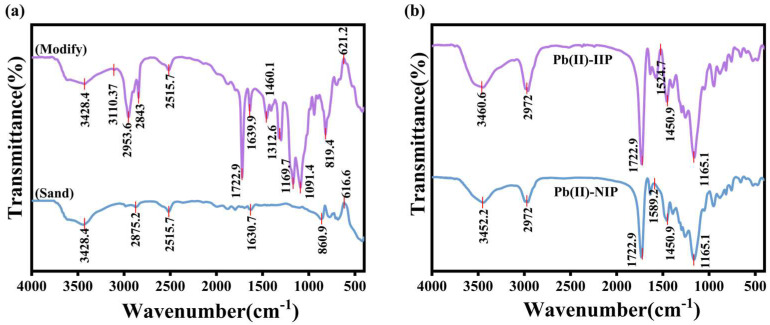
FTIR spectra of sand particles and modify sand particles (**a**) and MS/Pb(II)-IIP and MS/Pb(II)-NIP (**b**).

The surface of sand particles contains groups such as Si-OH, which exhibit significant infrared absorption. The sand particle spectrum (Figure 5a) displays an absorption peak at 3428.4 cm^−1^, which can be traced back to the stretching oscillation of -OH groups. At the same time, the -NH_2_ in KH550 remains on the surface of the sand particles, which can be observed at 3110.37 cm^−1^. The KH550 molecule contains alkyl chains, which exhibit characteristic infrared absorption after modification by the sand particles. Absorption peaks at 2953.6 cm^−1^ and 2843 cm^−1^ are indicative of the stretching vibrations of -CH_2_- and -CH_3_, respectively, introduced in KH550. There is a peak at 1722.9 cm^−1^ that is associated with the stretching vibration of the C=O. The absorption peaks at 1460.1 cm^−1^ and 1312.6 cm^−1^ are indicative of the bending vibrations of -CH_2_ and -CH_3_ in KH550. Similarly, the stretching vibration of Si-O bonds usually shows absorption peaks in the range of 1000–1100 cm^−1^. This peak appearing at 819.4 and 860.9 cm^−1^ is associated with the silica structure in the sand grains. For the KH550 modified sand, the peak at 621.2 cm^−1^ is associated with the Si-O-Si bending vibration. By comparing the two curves, it is obvious that the modified sand particles show new absorption peaks at some specific wave numbers, which correspond to the functional groups in the KH550, suggesting that the KH550 has been successfully modified to the surface of the sand particles. At the same time, the intensity and shape of some original peaks of the modified sand particles may also be changed, which reflects the altered chemical environment of the sand particle surface.

To identify the functional groups in Pb(II)-IIP and Pb(II)-NIP, FT-IR analysis was adopted. In Figure 5b, their peaks are the same in terms of shape and position, indicating that they belong to the same class of polymers [29]. The broad peaks observed at 3452.2 cm^−1^ and 3460.6 cm^−1^ are credited to -OH stretching vibrations. The peak at 2972 cm^−1^ is associated with -CH_2_ or -CH_3_ stretching vibrations, which come from the polymer backbone. The similar peak position but the difference in intensity at 1722.9 cm^−1^ is related to the stretching vibration of C=O. In Pb(II)-IIP, C=O is involved in the complexation reaction with Pb(II), which exhibits a different intensity of C=O peaks on the infrared spectrum from that of Pb(II)-NIP. A reading of 1165.1 cm^−1^ is associated with the stretching vibration of the C-O bond, originating from the chemical bonding in the polymer formed with the participation of AA. Pb(II)-IIP and Pb(II)-NIP have similar peak positions near 1589.2 cm^−1^ to 1450.9 cm^−1^, but there are differences in peak shapes, which are related to the functional groups in AA and AN. This reflects that the chemical environments of the linking groups, such as C-O-C or C-N, were changed during the blotting process because these groups were involved in the recognition and binding of Pb(II) during the synthesis of Pb(II)-IIP, suggesting that the blotting polymers have specific recognition sites that can interact with the template ions. By analyzing the infrared spectra of Pb(II)-IIP and Pb(II)-NIP, it can be speculated that during the synthesis of Pb(II)-IIP, the interactions of specific functional groups with Pb(II) lead to the formation of its unique structure, which results in the adsorption effect on Pb(II).

#### 3.1.4. SEM Analysis

**Figure 6:**
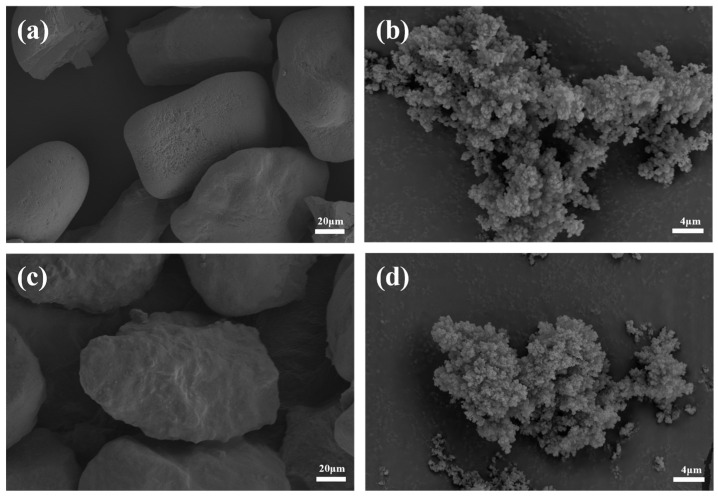
SEM images of sand particles and modify sand particles (**a**,**c**) and MS/Pb(II)-IIP: (**b**) after elution and (**d**) before elution.

The surface morphologies of the sand before and after modification, and of Pb(II)-IIP before and after template elution, were characterized by using SEM. From Figure 6a, it is evident that the surface of sand particles is relatively smooth, with irregular particle shapes. Sand particles of different sizes were piled up with each other, resulting in a tighter aggregation of particles. It can be observed from the electron microscope image in Figure 6c that the shape of the KH550-modified sand particles remains irregular, but the surface is rough, with obvious undulations, which is due to the chemical reaction of the KH550 molecules forming a coating layer on the surface of the sand particles and changing the surface properties. The modified sand particles exhibit relative looseness, with an increase in particle spacing.

Figure 6b,d display the three-dimensional network structure of the Pb(II)-IIP surface before and after elution, demonstrating that the elimination of template ions does not change the structure of the imprinted polymer. However, in terms of the overall structure, the imprinted polymer before elution in Figure 6d presents a more compact state. This tight structure is due to the strong interactions that happen between the template ions and the functional monomers during the synthesis process. The imprinted polymer in Figure 6b is slightly looser after elution than before elution, which is because the template ions are removed during the elution process, resulting in a certain degree of alteration of the polymer surface. In addition, the surface of the imprinted polymer (Figure 6d) was relatively flat and smooth before elution, while the surface of the imprinted polymer (Figure 6b) became rough after elution. This change is because the template ions leave many imprinted cavities on the polymer surface after elution. This structural change is the key to the ability of ion-imprinted polymers to effectively recognize and adsorb target ions in practical applications. When the target ions match the size, shape, and chemical environment of the cavities, they can enter the cavities more effectively and interact with the internal functional groups, thus achieving specific adsorption. This spatial matching effect is similar to the “lock and key” relationship, which enables ion-imprinted polymers to have a relatively high selectivity for target ions while having relatively weak adsorption for other ions.

#### 3.1.5. N_2_ Adsorption–Desorption Analysis

**Figure 7:**
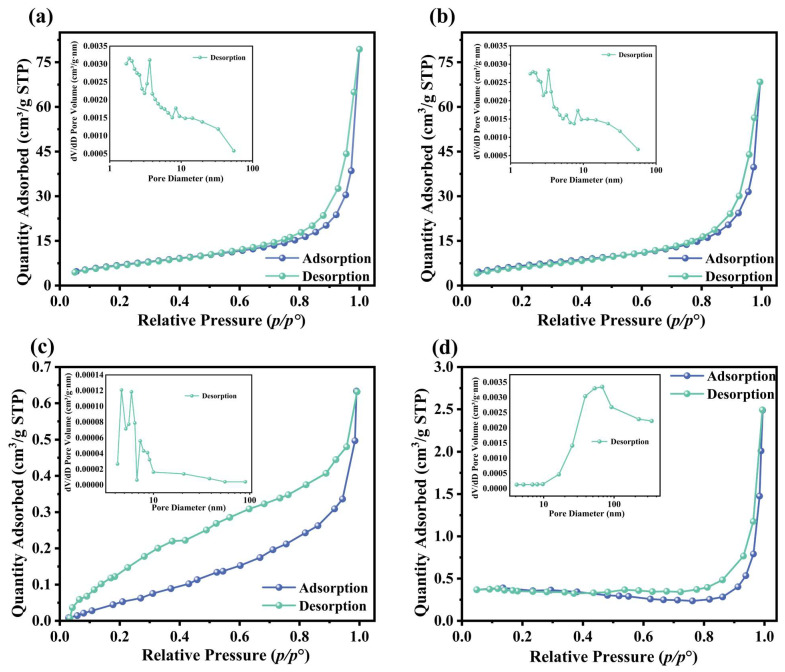
N_2_ adsorption–desorption isotherms: (**a**) MS/Pb(II)-IIP; (**b**) MS/Pb(II)-NIP; (**c**) sand particles; and (**d**) modify sand particles.

Figure 7 displays the N_2_ adsorption–desorption isotherms of MS/Pb(II)-IIP and MS/Pb(II)-NIP, while Table 1 displays the surface area per unit mass as well as the pore structure. The adsorption of MS/Pb(II)-IIP (Figure 7a) increases quickly at low relative pressures (*p*/*p*^0^ < 0.1), which suggests that the material has a microporous structure and is mostly adsorbed in a single layer at low pressures. The adsorption and desorption curves show a clear hysteresis loop at relative pressures of about 0.4–0.5, which is usually associated with mesopores. At high relative pressures (*p*/*p*^0^ > 0.9), the increase in adsorption tends to level off, suggesting that the material might have a macroporous structure. The overall adsorption amount of MS/Pb(II)-NIP (Figure 6b) is lower than that of MS/Pb(II)-IIP. The adsorption and detachment curves also show hysteresis loops at relative pressures of about 0.4–0.5, but the width and height of these loops are smaller than those of MS/Pb-IIP. The width and height of the hysteresis loops were smaller than those of MS/Pb(II)-IIP, indicating that the mesoporous structure was relatively small. This is because the holes in MS/Pb(II)-IIP are able to specifically adsorb certain molecules, and this specific adsorption is based on the shape, size, and chemical function match between the holes and the target molecules. The presence of such specific adsorption sites allows the imprinted polymers to trap gas molecules more efficiently during adsorption, further increasing their specific surface area. The adsorption performance of the material can be inferred from the adsorption–desorption curves in Figure 7a,b. The larger hysteresis loop indicates that the material has more mesopores, which is favorable for the diffusion and adsorption of adsorbate molecules in the pores. The magnitude of the adsorption amount reflects the adsorption capacity of the material, and a higher adsorption amount means that the material has more active sites for adsorption. The adsorption–desorption curves in Figure 7c,d all show the typical type IV isotherms. The hysteresis loops of the adsorption–desorption curves in Figure 7d are more pronounced than those in Figure 7c, which may imply that the modification process has altered the pore structure of the sand grains, possibly increasing the number or size of mesopores. Observing the adsorption amount of Figure 7c,d at low relative pressure, the adsorption amount of Figure 7d is significantly higher than that of Figure 7c. The modified sand particles have a larger specific surface area and, for the carrier of ion-imprinted materials, a larger specific surface area means more active sites available for adsorption, which is conducive to improving the adsorption capacity of ion-imprinted materials for the target ions.

### 3.2. Static Adsorption Studies

#### 3.2.1. Influence of Multiple Aspects Regarding the Adsorption Process

Influence of Contact Time

**Figure 8:**
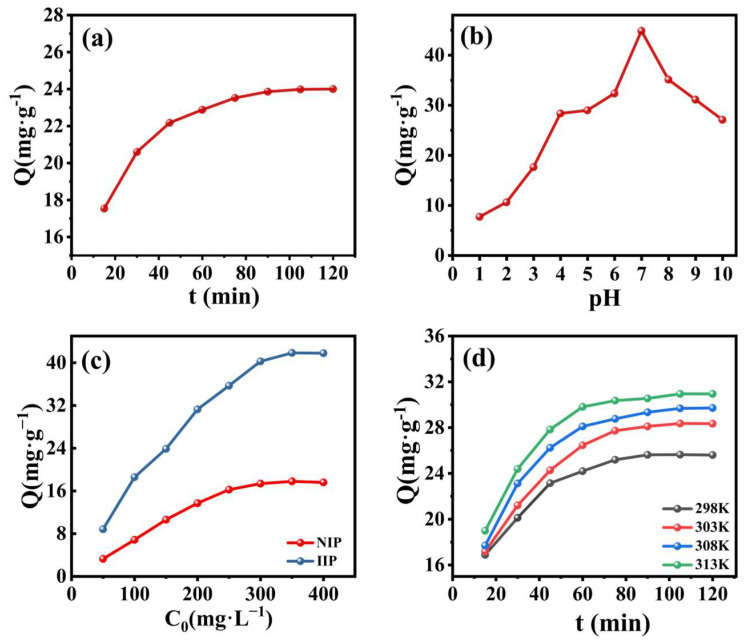
Influence of diverse factors on adsorption: (**a**) time; (**b**) pH; (**c**) initial solution concentration (experimental parameters: pH = 7; sample volume: 10 mL; temperature: 298 K; C_0_ = 100 mg L^−1^; and time: 105 min); and (**d**) temperature.

In the experiment examining the impact of contact time on adsorption performance, Figure 8a demonstrates a gradual increase in Pb(II) adsorption by the polymer as the adsorption time lengthens. The adsorption rate also demonstrates an upward trend before stabilizing, reaching equilibrium at 105 min as the polymer’s surface adsorption sites gradually fill up. At the onset of the adsorption process, the contact time is short. Pb(II) and the imprinted polymer have not yet fully interacted and reacted, thus resulting in a low adsorption amount. However, the polymer surface has many free adsorption sites, which Pb(II) in solution can quickly bind to. This leads to an increase in both the adsorption rate and the adsorption capacity. The ion-imprinted polymer possesses specific recognition sites for the target ions, and strong interactions, including ionic bonding, ligand bonding, and hydrogen bonding, facilitate the rapid adsorption of the ions onto the polymer surface. As the duration of adsorption prolongs, the adsorption sites located on the polymer surface are gradually occupied. The ions then need to permeate into the inner pores of the polymer to seek out the unoccupied adsorption sites. This process is relatively slow, which leads to a progressive decline in the adsorption rate, yet the adsorption capacity continues to increase. At this stage, the extension of time facilitates the diffusion of ions within the polymer, enabling more ions to penetrate the inner pores of the polymer, thus increasing the adsorption capacity. Once the adsorption time reaches a sufficient length, the adsorption sites both on the polymer’s surface and inside reach a state of complete occupancy, resulting in a dynamic equilibrium between adsorption and desorption, and the adsorption capacity no longer experiences significant changes as time increases. At this point, the impact of further prolonging the adsorption time on increasing the adsorption capacity is very limited.

Influence of pH

The pH of the Pb(II) solution constitutes a crucial factor during the adsorption process of the imprinted polymer. To investigate this, Pb(II) solutions of the same concentration with pH gradients of 1–10 were prepared to study the influence of solution acidity on the adsorption of Pb(II) by Pb(II)-IIP. The findings are shown in Figure 8b. One can observe the adsorption capacity of Pb(II)-IIP towards Pb(II) tending to increase as the pH rises and reaching its maximum when the pH = 7. When the pH was lower than the optimum level, the ionization of -COOH was hindered, which led to a reduction in the number of negative charges and a weakening of the electrostatic influence. At pH = 7, the ionization of -COOH is moderate, allowing the polymer to carry a moderate amount of negative charge. The negative charges can form electrostatic interactions with Pb(II) ions carrying a positive charge, thereby enhancing the adsorption efficiency towards Pb(II). However, the adsorption declined gradually as the pH continued to rise. This might be ascribed to the formation of Pb(OH)_2_ and its changed chemical and spatial structure, which diminishes its binding ability with the polymer and impedes the uptake of Pb(II). Pb(II)-IIP exhibits the optimal adsorption performance for Pb(II) at pH = 7.The theoretical optimal pH can be analyzed from the perspective of the precipitation equilibrium of Pb(II). Under the critical state where precipitation is about to form but has not yet formed in large quantities, through the solubility product constant *K_sp_* (Equation (7)), combined with the known initial concentration of Pb(II) and the ion product constant of water *K_w_* (Equation (8)), the theoretical pH = 6.25 can be obtained.

(7) $$K_{sp}=\left[{Pb}^{2+}\right][{OH}^{-}{]}^{2}$$

(8) $$K_{w}=\left[H^{+}\right][{OH}^{-}]$$

As functional monomers, AA and AN have a complexation effect with Pb(II). In the Pb(II)-containing solution that was originally in a certain equilibrium state, when the amount of Pb(II) decreases, it will be found that the pH of the solution increases compared with that before and shifts towards the alkaline direction when the pH of the solution is detected subsequently. Therefore, the optimum pH = 7 in this experiment.

Influence of Initial Concentration

Pb(II) solutions with concentrations ranging from 50 mg·g^−1^ to 400 mg·g^−1^ were added to MS/Pb(II)-IIP and MS/Pb(II)-NIP. Subsequently, the mixtures attained adsorption equilibrium and were then centrifuged to obtain the supernatant. This was done to explore the impact of varying concentrations on the adsorption properties, which is illustrated in Figure 8c. The adsorption capacity of MS/Pb(II)-IIP was increased with increasing concentration of the Pb(II) solution. Meanwhile, a substantial number of target ions came into contact with the adsorption sites located on the polymer surface, and the adsorption driving force was strengthened. In the initial phase, the adsorption rate is quite rapid and the adsorption capacity climbs quickly. With the progress of adsorption, the adsorption sites on the polymer are occupied gradually. When most of the adsorption sites are occupied, the increased rate of adsorption capacity gradually slows down with the continuous increase in ion concentration. Eventually, when all the specific adsorption sites are saturated, the adsorption reaches equilibrium and the adsorption capacity no longer changes significantly with increasing ion concentration. The adsorption efficiency of MS/Pb(II)-IIP towards Pb(II) was remarkably superior to that of MS/Pb(II)-NIP under various initial concentration conditions. The saturated adsorption amounts of MS/Pb(II)-NIP and MS/Pb(II)-IIP were 17.78 mg·g^−1^ and 41.83 mg·g^−1^. The remarkable adsorption property for Pb(II) is attributable to the particular binding sites located upon the surface of the eluted template ions as well as the imprinted pore size, which is geometrically matched. The imprinted polymer is prepared using Pb(II) as a template, giving rise to the generation of specific recognition sites within the polymer that are congruent with Pb(II) in terms of spatial structure, size, and chemical function. When Pb(II) is present in the solution, these sites are highly selective to accurately recognize and bind Pb(II), leading to enhanced adsorption of Pb(II) by the polymer. The non-imprinted polymer surface lacks such specific recognition sites.

Influence of Temperature

The experiment aimed to explore how temperature affected the adsorption efficiency of Pb(II) between 298 K and 313 K. The result is shown in Figure 8d, demonstrating that the adsorption capacity is able to reach 32.95 mg·g^−1^ in 120 min when the temperature reaches 313 K. From a thermodynamic perspective, temperature alterations influence the molecular thermal motion, which subsequently impacts the adsorption and desorption rates of the adsorption process. The adsorption capacity increased with the elevation of the temperature of the adsorption process in the range between 298 K and 313 K. The impact of temperature on the adsorption process varies in different studies. Therefore, thermodynamic property studies were conducted. The following equation calculates the thermodynamic parameters [41]:

(9) $$∆G^{0}=-RTlnK_{d}$$

(10) $$lnK_{d}=-\frac{∆H^{0}}{RT}+\frac{∆S^{0}}{R}$$

where *K_d_* represents the equilibrium constant, *R* stands for the ideal gas constant, *∆G*^0^ (kJ·mol^−1^) denotes the Gibbs free energy, *∆H*^0^ (kJ·mol^−1^) indicates the enthalpy change, ∆S^0^ (J·mol^−1^·k^−1^) refers to the entropy change, and *T* (K) corresponds to temperature.

Table 2 presents the thermodynamic parameters of the adsorption of Pb(II) by MS/Pb(II)-IIP. The negative values of *∆G*^0^ at all temperatures suggest that the adsorption process occurs spontaneously within these temperature ranges [42]. As the thermal condition rises within the range of 298 K to 313 K, the Δ*G*^0^ becomes slightly more negative, suggesting that the increase in temperature exerts a certain facilitating effect on the spontaneity of the reaction. Δ*S*^0^ indicates that the disorder of the system increases during the process [43], and Δ*H*^0^ indicates that the process is an exothermic reaction [44].

#### 3.2.2. Adsorption Isotherm

**Figure 9:**
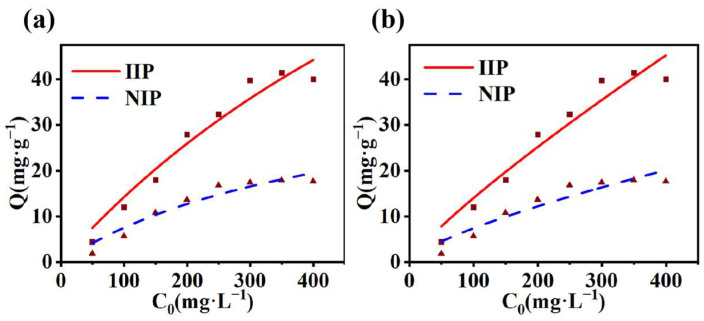
Adsorption isotherm curves of MS/Pb-IIP and MS/Pb-NIP: (**a**) Langmuir isotherm and (**b**) Freundlich isotherm.

An isothermal adsorption line is a curve that represents the relationship between the quantity of adsorbate adsorbed by an adsorbent and the equilibrium concentration of the adsorbate under a specific temperature condition. The Langmuir model (Equation (11)) and the Freundlich model (Equation (12)) are the ones commonly adopted as isothermal adsorption models. To provide a more comprehensive illustration of the adsorption behavior of MS/Pb(II)-IIP towards Pb(II), the Langmuir isotherm model, as well as the Freundlich isotherm model were employed for fitting. The fitting results are depicted in Figure 9, and the specific values of the relevant parameters are enumerated in Table 3. The R^2^ for MS/Pb(II)-IIP and MS/Pb(II)-NIP fitted by the Langmuir adsorption isotherm model were 0.95 and 0.92, respectively. The R^2^ for MS/Pb(II)-IIP and MS/Pb(II)-NIP fitted by the Freundlich adsorption isotherm model were 0.94 and 0.88, respectively. In the Langmuir fitting, the maximum adsorption of MS/Pb(II)-IIP was much higher than that of MS/Pb(II)-NIP. This also suggests that the monolayer coverage is the most important part of the adsorption process for Pb(II) from water by MS/Pb(II)-IIP. In the Freundlich fit, the adsorption of MS/Pb(II)-IIP became more significant as the concentration went up. This shows that ion-imprinted polymers also adsorb better when they are stacked in multiple layers.

Overall, MS/Pb(II)-IIP exhibited superior adsorption performance compared to MS/Pb(II)-NIP, regardless of whether it was monolayer adsorption (Langmuir model) or multilayer adsorption (Freundlich model). This superiority could be ascribed to the existence of specific recognition sites within the ion-imprinted polymers, which enabled them to bind adsorbates more efficiently.

(11) $$Q_{e}=\frac{Q_{m}C_{e}K_{L}}{1+K_{L}C_{e}}$$

(12) $$Q_{e}=K_{F}C_{e}^{1/n}$$

*Q_e_* and *Q_m_* are the equilibrium adsorption amount and maximum adsorption amount (mg·g^−1^), the solubility of Pb(II) in solution when *C_e_* adsorption reaches equilibrium (mg·L^−1^), *K_L_* is the constant related to the adsorption strength, and *K_F_* and n are the Freundlich’s constants, which are correlated with the adsorption amount and adsorption strength.

#### 3.2.3. Adsorption Kinetic

In the kinetic study of the adsorption process, a quasi-first-order kinetic model (Equation (13)) and a quasi-secondary kinetic model (Equation (14)) were employed for the analysis of the experimental data.

(13) $$\ln\left(Q_{e}-Q_{t}\right)=lnQ_{e}-k_{1}t$$

(14) $$\frac{t}{Q_{t}}=\frac{k_{2}}{Q_{e}^{2}}+\frac{t}{Q_{e}}$$

where *Q_e_* (mg⋅g^−1^) represents the adsorbed amount when adsorption equilibrium is reached, while *Q_t_* (mg⋅g^−1^) stands for the adsorbed amount at time *t*. *k*_1_ refers to the rate constant of the quasi-first-order kinetic equations, and *k*_2_ is the rate constant of the quasi-secondary kinetic equations.

**Figure 10:**
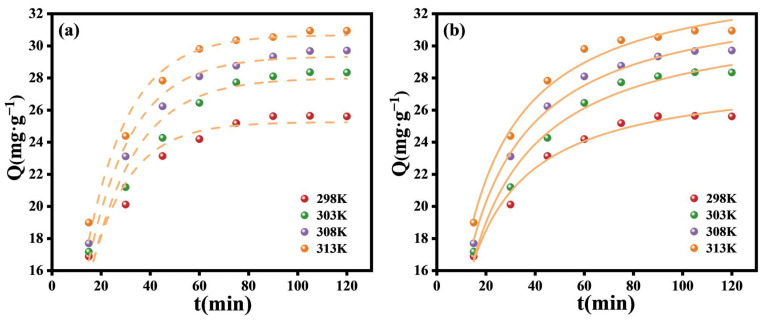
Quasi-first-order (**a**) and quasi-second-order kinetic model (**b**) fitting at different reaction temperatures.

Figure 10a presents the adaptation of the quasi-first-order kinetic model at different temperatures, revealing a certain degree of linear relationship in each temperature interval. This indicates that the adsorption process has a mechanism that aligns with the description of quasi-first-order kinetics. Figure 10b presents the fitting outcomes regarding the quasi-secondary kinetic model at different temperatures. In comparison with the quasi-first-order kinetic model, the R^2^ of the quasi-second-order kinetic model is closer to one at all temperatures, but there is not much difference between the two. Therefore, the adsorption process can be regarded as the consequence of the combined action of quasi-first-order and quasi-second-order kinetics. At the start of the adsorption process, the adsorbent surface has more empty spots. The adsorbate molecules are more likely to diffuse and bind to these spots because their concentrations are different, which fits with the idea that adsorption works in a way that is similar to primary kinetics. As the adsorption process proceeds, the chemical reactions occurring between the adsorbate and the reactive positions on the adsorbent surface become more intense, resulting in the quasi-second-order kinetic mechanism dominating the subsequent adsorption process. And the kinetic model parameters are shown in Table 4.

### 3.3. Adsorption Selectivity

**Figure 11:**
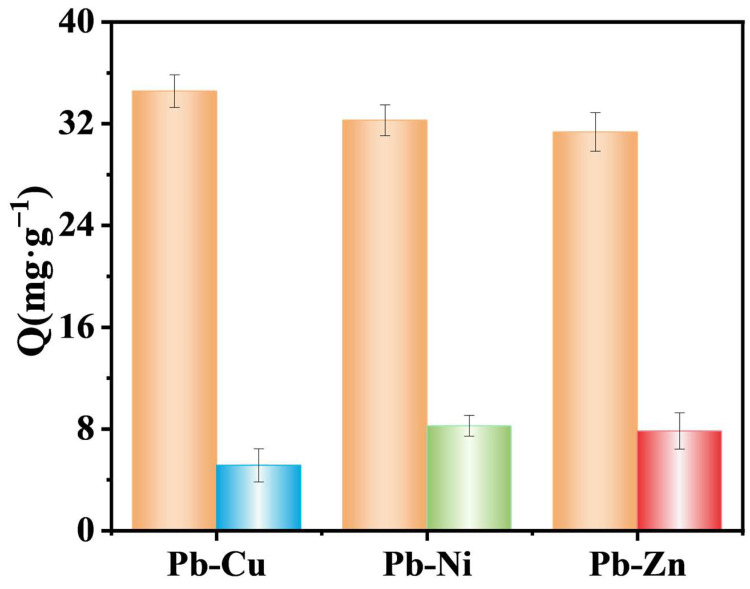
Selective adsorption of Pb(II) by MS/Pb(II)-IIP.

In order to investigate the effect of ion-imprinted polymer (IIP) on the adsorption properties of Pb(II) in the presence of coexisting cations, mixed solutions (0.5 mmol/L) containing the same concentrations of Pb-Cu, Pb-Ni, and Pb-Zn were prepared, and then the adsorbent of ion-imprinted polymer (IIP) was added into the above different mixed solutions, respectively. The experimental data that have been obtained are displayed in Figure 11. In an identical environment, the adsorption of Cu(II) by the composites in the Pb-Cu ion mixing solution was determined to be negligible. The adsorption of Ni(II) by the composites in the Pb-Ni ion mixing solution was less than half that of Pb(II), while the ion adsorption of Zn(II) by the composites in the Pb-Zn ion mixing solution was less than half that of Pb(II). The findings reveal that the fabricated composites display selective adsorption towards Pb(II). Despite the similar charge and size of competing ions with Pb(II), as well as their high affinity for oxygen atoms in acrylic acid and acrylonitrile, IIP exhibits a remarkable selectivity towards Pb(II). The adsorption selectivity factors of MS/Pb(II)-IIP materials are listed in Table 5.

### 3.4. Reuse Experiment of MS/Pb(II)-IIP

The recyclability of adsorbents represents a key determinant of their economic value [45]. Checking how many times the material can be used gives us information about how stable MS/Pb(II)-IIP is during multiple cycles of adsorption and desorption, which is an important part of figuring out if the material is commercially viable.

**Figure 12:**
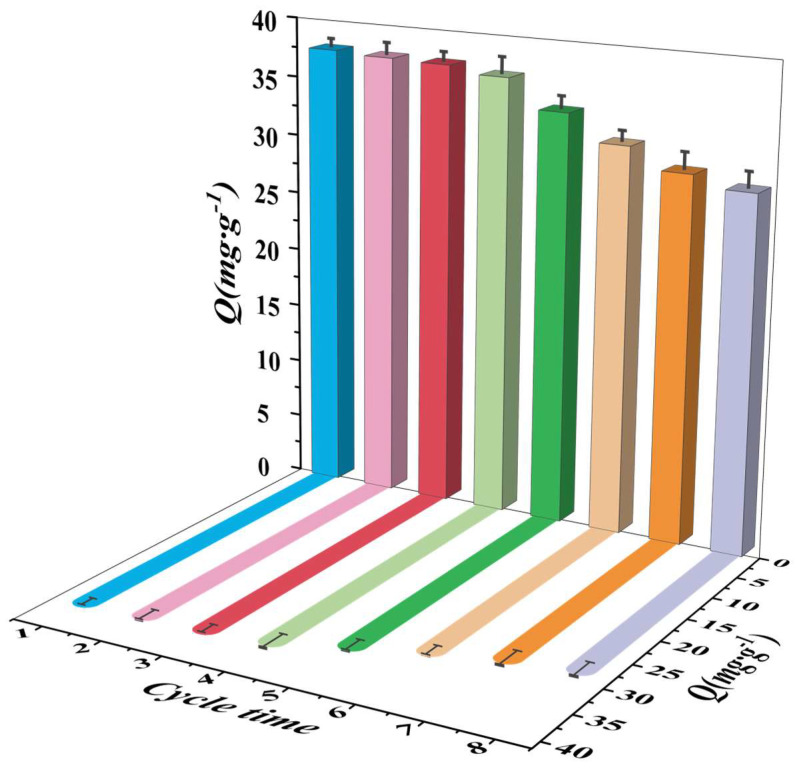
Reuse experiment of MS/Pb(II)-IIP.

After the initial adsorption experiments, the subsequent adsorption–desorption cycle was carried out by using hydrochloric acid (HCl) at a concentration of 1 mol/L as a desorbent at a constant temperature of 25 °C for 30 min at an oscillation rate of 150 rpm. Figure 12 presents the experimental results, observing a gradual decline in the adsorption capacity of MS/Pb(II)-IIP on Pb(II) after several cycles. This phenomenon can be explained by the use of hydrochloric acid as a desorbent, which caused some damage to the imprinted sites on the surface of the composites during the desorption process. However, after eight cycles, MS/Pb(II)–IIP could only adsorb 29.76 mg·g^−1^ of Pb(II), which means that the adsorption efficiency stayed above 79%. Despite hydrochloric acid damaging the complex material’s surface, MS/Pb(II)-IIP demonstrated a high degree of reusability. This is significant in practical applications, demonstrating the excellent reproducibility of MS/Pb(II)-IIP. This implies that a simple desorption operation can reuse the adsorbent for adsorption, thereby lowering costs and enhancing economic efficiency. Furthermore, it can predict the material’s service life, serving as a reference for the material replacement cycle in practical applications, thereby ensuring the efficiency and stability of the treatment process. Table 6 is a comparison of MS/Pb(II)-IIP with other adsorbents for Pb(II) adsorption.

### 3.5. Analysis of Imprinting Mechanism

**Figure 13:**
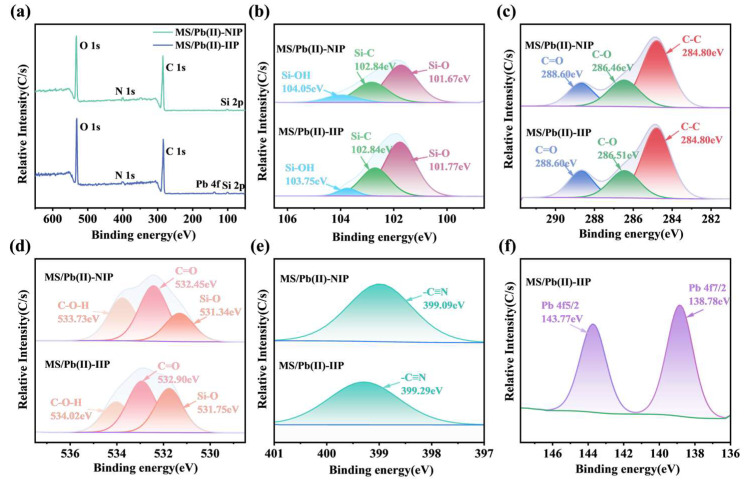
XPS spectra: (**a**) full spectrum; (**b**) MS/Pb(II)-NIP and MS/Pb(II)-IIP Si 2p spectrum; (**c**) MS/Pb(II)-NIP and MS/Pb(II)-IIP C 1s spectrum; (**d**) MS/Pb(II)-NIP and MS/Pb(II)-IIP O 1s spectrum; (**e**) MS/Pb(II)-NIP and MS/Pb(II)-IIP N 1s spectrum; and (**f**) MS/Pb(II)-IIP Pb 4f spectrum.

We performed XPS analysis to deeply investigate the surface properties and imprinting mechanism of MS/Pb(II)-IIP and MS/Pb(II)-NIP [50]. XPS can supply crucial information regarding the chemical state and electronic environment of the surface elements within the materials. Figure 13a: the XPS full spectrum demonstrates the detection of both MS/Pb(II)-IIP and MS/Pb(II)-NIP, Si from the sand grains and C, N, and O from the functional monomers. Additionally, the MS/Pb(II)-IIP presented characteristic peaks corresponding to the Pb element, aligning with our preparative system and further confirming the elements’ successful participation in the polymer construction process. Figure 13b the presence of AA, AN, and Pb(II) in MS/Pb(II)-IIP causes AA to form a bond with Si-O on the sand grains’ surface during polymerization. This bonding modifies the electron cloud density in the surroundings and displaces the peak position corresponding to Si-O, moving it towards higher binding energy. Meanwhile, AN forms more Si-C with the sand grain surface, enhancing Si-C in MS/Pb(II)-IIP compared to MS/Pb(II)-NIP. Figure 13c: Pb(II) changes the chemical environment around C in the polymer. This causes the electron cloud density of C interacted with the template ions to decrease, the binding energy to rise, and the peak position to move toward the high binding energy. Figure 13d: upon the interaction between C=O and Pb(II) in MS/Pb(II)-IIP, the electron cloud concentration around them is reduced, and the peak shifts towards a higher binding energy. The polymerization reaction reduces -OH, which in turn reduces the peak intensity. Some of the N from AN in Figure 13e interacts with Pb(II). This makes the electron cloud around the N less dense and moves the peak toward higher binding energies. The sharpness of the Pb(II) peak in Figure 13f is ascribable to the relatively uniform environment of Pb(II) in polymers. It revolves around the specific recognition site and engages in a single interaction with the other atoms.

## 4. Conclusions

In this study, we selected and combined sand particles, a common yet innovative carrier for applications in this field, with specific functional monomers (acrylonitrile and acrylic acid) and template ions (lead ions) to create composites with unique adsorption properties and recognition functions for Pb(II). We conducted characterization using UV–vis, SEM, BET, FTIR, XPS, and other test methods to confirm the imprinting incidence and investigate the adsorption mechanism. We constructed accurate adsorption kinetics and adsorption isotherm models by studying the kinetic and thermodynamic properties of MS/Pb(II)-IIP, confirming that its adsorption behavior conforms to the Langmuir adsorption isotherm model based on monomolecular layers, the quasi-primary and quasi-secondary kinetic equations, and that the adsorption process is an entropy-increasing spontaneous process. It showed good selectivity in selective adsorption experiments, and the adsorption efficiency remained at 79% after eight adsorption–desorption cycle tests.

## Figures and Tables

**Table 1:** Surface area per unit mass and pore structure parameters.

Sample	Specific Surface Area (m^2^·g^−1^)	Pore Volume (cm^3^·g^−1^)	Average Pore Diameter (nm)
MS/Pb(II)-IIP	25.63	0.121	19.160
MS/Pb(II)-NIP	23.48	0.104	17.180
Sand particles	0.760	0.0386	9.800
Modify Sand particles	1.577	0.0979	10.711

**Table 2:** Thermodynamic parameters of MS/Pb(II)-IIP.

∆G^0^ (kJ·mol^−1^)				∆*S*^0^ (J·mol^−1^·k^−1^)	∆*H*^0^ (kJ·mol^−1^)
298 K	303 K	308 K	313 K		
−4.083	−3.941	−4.151	−4.483	7.000	−1.994

**Table 3:** Langmuir and Freundlich isotherm parameter.

Adsorbent	Langmuir Isotherm			Freundlich Isotherm		
	K_L_	Q_m_ (mg·g^−1^)	R^2^	K_F_	1/n	R^2^
XS/Pb-IIP	1.04 × 10^−3^	150.34	0.95	0.282	0.847	0.94
XS/Pb-NIP	2.18 × 10^−3^	41.79	0.92	0.263	0.724	0.88

**Table 4:** MS/Pb(II)-IIP kinetic modelling parameters.

T (K)	Quasi-First-Order			Quasi-Second-Order		
	Q_e_, cal mg·g^−1^	k_1_ min^−1^	R^2^	Q_e_, cal mg·g^−1^	k_2_ min^−1^	R^2^
298 K	25.53	0.060	0.90	26.02	0.033	0.93
303 K	27.95	0.053	0.94	28.82	0.022	0.97
308 K	29.32	0.055	0.98	30.25	0.022	0.96
313 K	30.65	0.058	0.97	31.62	0.023	0.98

**Table 5:** Assignment coefficients k_d_ and relative selectivity coefficients k for MS/Pb(II)-IIP.

	k_d_ (Pb^2+^)	k_d_ (M^2+^)	k
Pb(II)/Cu(II)	0.533	0.058	9.19
Pb(II)/Zn(II)	0.462	0.089	5.19
Pb(II)/Ni(II)	0.479	0.095	5.04

**Table 6:** Comparison of adsorption capacity of various imprinted materials for Pb(II).

Carrier	Adsorption Capacity (mg·g^−1^)	Capacity Retention (%)	Ref.
PAN nanofiber	43.1	69.91 (6 cycles)	[46]
—	25.3	60 (10 cycles)	[47]
MWCNTs	18.09	95 (5 cycles)	[48]
Fe_3_O_4_	21.32	97 (21 cycles)	[49]
modified sand particles	41.83	79 (8 cycles)	This work

## Data Availability

The data presented in this study are available on request from the corresponding author.
